# Innate immunity in fungi: Is regulated cell death involved?

**DOI:** 10.1371/journal.ppat.1010460

**Published:** 2022-05-19

**Authors:** Maria Laura Gaspar, Teresa E. Pawlowska

**Affiliations:** School of Integrative Plant Science, Cornell University, Ithaca, New York, United States of America; McGill University, CANADA

## Overview

Innate immunity is an ancient cell-autonomous property of eukaryotes that allows them to regulate interactions with antagonistic microbes, including bacteria. While animal and plant innate immunity systems are relatively well understood [1,2], innate defenses in fungi are only beginning to be unraveled. Both animal and plant immune systems consist of surveillance, signal transduction, and response modules, which exhibit remarkable functional similarities and are clearly products of convergent/parallel evolutionary trajectories [3,4]. Among others, these similarities include **regulated cell death** (**RCD**) of infected cells as an ultimate mechanism for eliminating microbial intruders. Unlike accidental cell death, RCD is governed by a set of genetically encoded procedures for targeted cell removal and, depending on specific stimuli, can proceed along several distinct pathways manifested by diverse biochemical and morphological hallmarks [5].

It is unclear whether the convergent patterns in animal and plant innate defenses are a consequence of (i) adaptation to similar selective forces exerted by microbial antagonists; (ii) underlying physical, biochemical, or developmental constraints imposed by solutions needed to combat invading microbes; (iii) stochasticity of genetic drift; or (iv) a combination of all these 3 forces [6]. Nonetheless, it is not unreasonable to expect that elements of innate immunity have also evolved in fungi.

The limited knowledge about antimicrobial defenses in fungi could be attributed to the common perception that fungi depend on secondary metabolites to suppress bacterial antagonists [7]. Indeed, secondary metabolites, which are nonessential bioactive compounds of low molecular weight, play an outsized role in warfare and defense of some fungi, particularly Dikarya [8]. However, other fungi, including those representing early divergent lineages, such as Mucoromycotina, do not produce extensive repertoires of secondary metabolites and seem to rely on mechanisms resembling innate immunity of animals and plants for protection of their cells from bacterial invasions [9], as we detail in the subsequent sections.

Given convergent origins of animal and plant antimicrobial defenses, the existence of innate immunity in fungi is not surprising. Its architecture is likely a product of both, a close phylogenetic relation of fungi and animals, which are united in the supergroup Opistokonta, and physical constrains of a modular growth habit and rigid walls enveloping fungal cells, both features shared with plants. Because of these conditions, fungi are expected to share molecular underpinnings of their immune responses with animals, while being able to mount them in most vegetative cells of their bodies, like plants and in contrast to animals, which rely on specialized immune cells, phagocytes.

In this Pearl, we outline the convergently evolved innate immune modules of animals and plants, argue that antibacterial defenses of fungi exhibit hallmarks of innate immunity, explore the types of RCD in fungi, hypothesize that RCD is one of the defensive responses in fungi, present a model system to test this hypothesis, and consider the significance of such research for developing novel antifungal therapies.

## 1. Innate immunity surveillance systems

Animals and plants use **pattern recognition receptors** (**PRRs**) to sense conserved structural components of microbial cells, referred to as **microbe-associated molecular patterns** (**MAMPs**), as well as host-derived **damage-associated molecular patterns** (**DAMPs**) [1,2]. In animals, PRRs survey both extra- and intracellular environments and, among others, include Toll-like receptors, C-type lectin receptors, and **nucleotide-binding oligomerization domain (NOD)-like receptors** (**NLRs**) [1]. These PRRs are collectively capable of detecting ligands such as **peptidoglycan** (**PGN**), flagellin, lipopolysaccharides, and beta-glucans [1]. In plants, PRRs sense a similar set of extracellular MAMPs [2]. Despite this functional commonality, plant PRRs comprise receptor-like kinases and receptor-like proteins that differ structurally from animal PRRs [2]. Importantly, several classes of animal and plant PRRs include domains containing a varying number of **leucine-rich repeat** (**LRR**) motifs for ligand perception [1,2].

In contrast to animals and plants, fungi do not seem to harbor extracellular PRRs with LRR motifs [7]. Instead, as demonstrated in *Candida albicans*, they rely on LRR domains of adenylyl cyclases for sensing bacterial PGN, which is somewhat analogous to LRR domain-mediated PGN perception by mammalian NLRs [10]. Notably, with exceptions including Saccharomycotina and early divergent Mucoromycotina, most fungal genomes encode large and variable repertoires of up to 200 NLR-like proteins [11–13]. Fungal NLR-like proteins have a typical tripartite domain organization shared with animals and plans, with a central nucleotide-binding domain flanked by a amino-terminal effector domain and a carboxyl-terminal autoinhibitory/ligand-binding domain, often composed of repeat motifs. Importantly, instead of LRR motifs typical for animal and plants NLRs, fungal NLR-like proteins harbor ankyrin (ANK), WD (tryptophan- and aspartic acid-containing repeat), or tetratricopeptide (TRP) motifs at their carboxyl termini. Similarly, the diversity of effector domains at the amino termini of fungal NLR-like proteins far exceeds diversity levels found in animals and plants. As we discuss later, some of these NLR-like proteins mediate RCD resulting from vegetative/heterokaryon incompatibility in conspecific nonself-recognition reactions [14,15]. Fungi are also capable of sensing MAMPs other than PGN, including flagellin and lipooligosaccharides [16]. However, at present, the identity of the requisite sensors remains unknown.

## 2. Nonsuicidal innate immunity response modules

In animals and plants, binding of MAMP, DAMP, and effector ligands activates signaling cascades that lead to rapid transcriptional and nontranscriptional innate immunity responses. These responses converge on: (i) the release of reactive oxygen species (ROS) [17,18]; (ii) biosynthesis of antimicrobial peptides (AMPs) [19]; and (iii) deployment of extracellular traps (ETs) by phagocytes; in plants, ETs are produced in a continuous manner [20]. (i) **ROS** are highly reactive chemicals, such as superoxide anion and hydrogen peroxide, which kill pathogens by protein oxidation [17,18]. In phagocytes, oxidative burst is produced by the membrane-bound NADPH oxidase (NOX). In plants, ROS are generated as part of PRR-triggered immunity (PTI). Responses of PTI can be further amplified after microbial signals are perceived by plant NLRs, which activates effector-triggered immunity (ETI) [21]. ROS also play a role in interactions between fungi and bacteria in which fungi respond to bacterial antagonists with oxidative burst [9]. For example, in the early divergent mold *Rhizopus microsporus* challenged by *Mycetohabitans* sp., ROS accumulation is accompanied by upregulation of genes responsible for producing ROS and causing oxidative damage through prooxidant activity [9]. (ii) **AMPs** are small (approximately 100 amino acids) cysteine-rich molecules that disrupt bacterial cell envelopes [19]. In phagocytes, in addition to being synthesized in response to perception of microbial ligands, AMPs are expressed constitutively and stored in secretory granules as inactive precursors. Fungi also generate AMPs when challenged by bacteria [22]. For example, the coprophilous mushroom *Coprinopsis cinerea* produces cysteine stabilized αβ-defensins and GH24-type lysozymes when co-cultivated with *Bacillus subtilis* and *Escherichia coli* [22]. (iii) **ETs** are web-like structures composed of decondensed chromatin and granular proteins, including AMPs, which bind and kill bacteria [20]. Neutrophile extracellular traps (NETs) are prototypical ETs. In plants, ETs are produced continuously by border cells, which are specialized immune cells released from the root cap. While extracellular DNA is commonly found in fungal biofilms [23], as of yet, there is no evidence of ET formation by fungi.

While some antimicrobial responses of animals and plants are products of convergent evolution, these organisms rely also on mechanisms determined by their specific evolutionary heritage and constrains: phagocytosis [24] in animals and cell wall remodeling in plants [25]. **Phagocytosis** is absent from wall-enveloped cells of plants and fungi, as it involves folding of the plasma membrane around particles larger than 0.5 μm in diameter, including microorganisms, and internalizing them into a specialized vacuole called the phagosome, which fuses with the lysosome into the phagolysosome where the ingested particles are degraded. Phagocytosis is sometimes complemented by **autophagy**, a conserved homeostatic process unfolding in response to various cellular stresses, including microbial infection, starvation, or organelle damage [26]. Autophagy helps in clearing pathogens by engulfing them in a double-membraned vesicle, autophagosome. The autophagosome fuses with the lysosome, forming the autolysosome where cargo, including microbial cells, is degraded. Autophagy is an ancient process shared by animals, plants, and fungi [27]. In fact, autophagy was observed in the model ascomycete *Podospora anserina* confronted with antagonistic bacteria *Serratia fonticola* and *Serratia marcescens* [28]. However, it is not clear whether in this setting autophagy served as a mechanism to limit damages inflicted by bacteria or to eliminate the intruders.

In the absence of phagocytosis, **cell walls** serve as antimicrobial barriers in plants. Plant cell walls are composed of polysaccharides, including cellulose, pectin, and hemicelluloses [25]. Deposition of callose and phenolic compounds, such as lignin [25], which reinforce the wall, is part of plant PTI and can be further magnified by ETI [21]. Like in plants, fungal cells (hyphae) are also enveloped by cell walls composed of a scaffold of cross-linked polysaccharides, such as glucans, chitin, chitosan, and a matrix of proteins and mannans [29]. Fortification of the fungal cell wall as an antibacterial defense mechanism was implicated by transcriptional profiling of *R*. *microsporus* responses to antagonistic *Mycetohabitans* sp. [9]. Specifically, these patterns suggested a shift in the call wall composition by increasing the ratio of chitosan and 1,3-beta-glucan relative to chitin, which would ensure enhanced cell wall stability in the presence of bacterial chitinases.

Collectively, fungi respond to antagonistic bacteria like animals and plants by generating ROS and AMPs. Fortification of fungal cell walls mirroring plant responses to pathogens was also implicated. These patterns suggest that, while fungal surveillance modules dedicated to detection of bacteria may be distinct, response modules are analogous to those of animals and plants.

## 3. Suicidal innate immunity response modules

Both plants and animals employ RCD of infected cells to restrict the niche for proliferation of microbial intruders [30]. Sensing MAMPs and DAMPs by phagocyte NLRs activates them to oligomerize into **inflammasomes**, which serve as macromolecular scaffolds for recruitment and activation of the zymogen procaspase 1 [31]. Once activated, caspase 1 cleaves pro-inflammatory cytokines IL-1β and IL-18 into their bioactive forms, which are released through plasma membrane pores formed by **gasdermin D**. Gasdermin pores, together with the cell-surface NINJ1 protein [32], contribute to the disruption of plasma membrane integrity, leading to lytic RCD, **pyroptosis** [31]. Pyroptosis not only amplifies pro-inflammatory signals but also exposes surviving microbial cells to elimination by secondary phagocytes. Another notable type of pro-inflammatory death known as **nectroptosis** is a caspase-independent process induced, among others, by viral infections [33]. In necroptosis, the plasma membrane is disrupted by amyloid-like structures formed by oligomerization of the mixed lineage kinase domain–like (**MLKL**) protein monomers upon their translocation from the macromolecular necrosome to the membrane.

In plants, perception of microbial signals by NLRs, in addition to ETI, triggers the **hypersensitive response** (**HR**) [21]. Recent evidence suggests that, similar to the inflammasome’s role in vertebrate pyroptosis, HR is mediated by the action of the resistosome formed by oligomerization of NLR monomers [34]. Like the inflammasome, the resistosome mediates formation of pores that compromise cell membrane integrity. The program of HR in plant cells is carried out by proteases with caspase-like activities and metacaspases distantly related to animal caspases [35]. Notably, the spread of death signals is controlled by autophagy, which limits HR to the infected sections of plant tissues [36].

Clearly, animals and plants deploy several diverse RCD programs to combat infections. Whether fungi rely on RCD as an ultimate strategy for eliminating bacterial antagonists by sacrificing compromised hyphae remains unknown.

## 4. Is RCD involved in innate immunity of fungi?

Because clearing microbial invaders from animal and plant cells can be executed by several distinct RCD modes, like pyroptosis, necroptosis, or HR, these subroutines are often compared and contrasted with **apoptosis**, which is considered to be a prototypical form of RCD [5]. Apoptosis dates back to the domestication of the mitochondria by early eukaryotic cells [37] and generally is not involved in innate immunity responses of animals [5]. Apoptosis is defined by cytoplasmic shrinkage, chromatin condensation, nuclear fragmentation, and plasma membrane blebbing, culminating with the formation of membrane-bound apoptotic bodies subsequently degraded by phagocytes. In mammalian cells, the process of apoptosis can be initiated either by external signals (extrinsic pathway) or by perturbations in the cellular environment (intrinsic pathway). In response to intrinsic cellular stress, such as DNA damage, mitochondria release to the cytoplasm cytochrome *c*, a component of the electron transport chain. Once cytochrome *c* is detected by the apoptotic cytoplasmic protease-activating factor 1 (APAF1), APAF1 monomers oligomerize to form a multimeric complex of the **apoptosome**, which serves as a macromolecular platform for tethering and activation of zymogen procaspase 9 [38]. Caspase 9 cleaves and activates downstream executor procaspases that advance the cell death cascade by degrading various cellular targets, which results in morphological hallmarks of apoptosis.

**Fig 1:**
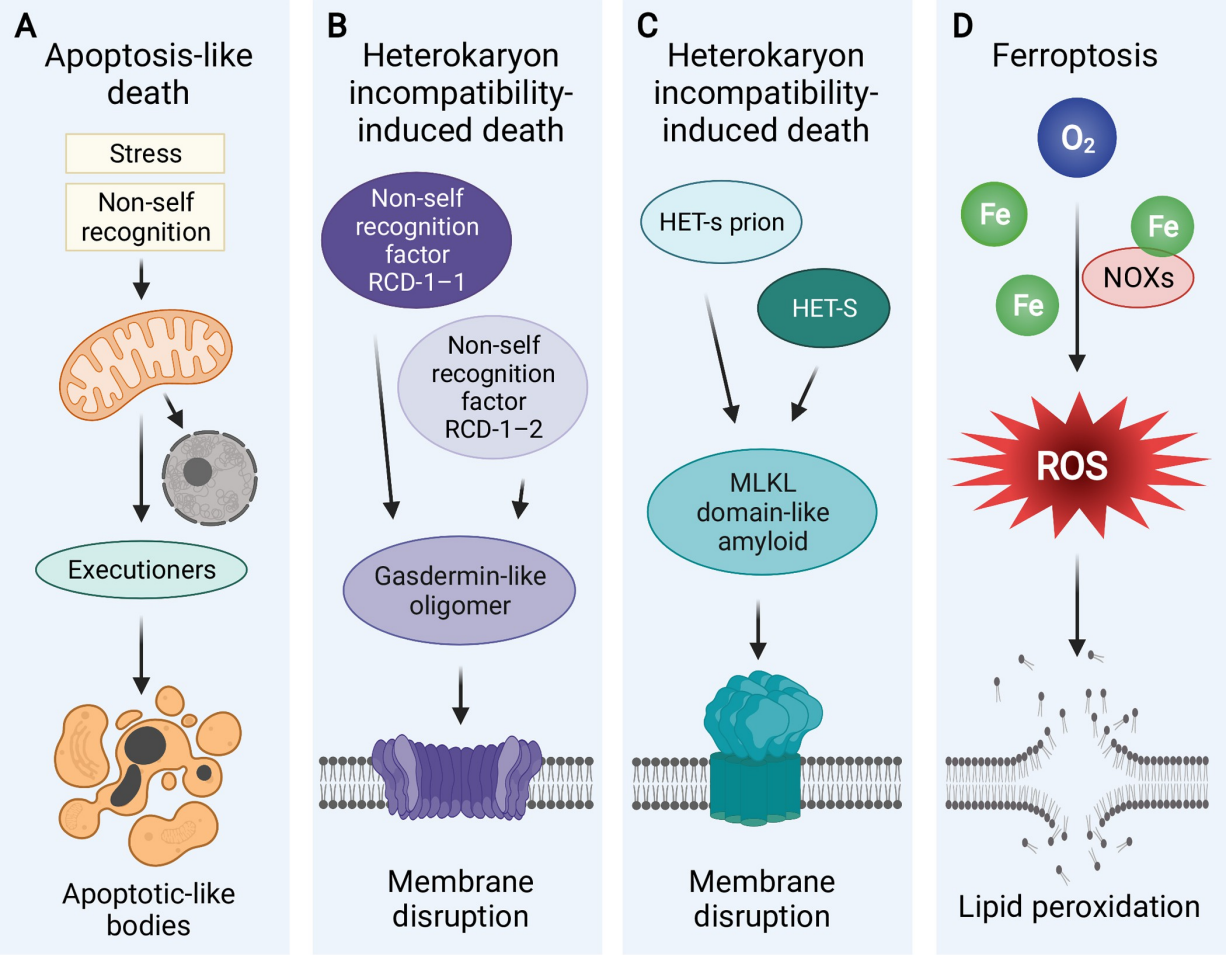
Types of RCD in fungi. **(A)** Apoptosis-like death elicited by stress [42] and vegetative/heterokaryon incompatibility [44], leading to degradation of various cellular targets and morphological hallmarks of apoptosis. **(B)** Heterokaryon incompatibility–induced death involving nonself-recognition factors RCD-1-2 and RCD-1-2 oligomerizing into the membrane disrupting gasdermin-like protein [47]. **(C)** Heterokaryon incompatibility–induced death involving the MLKL domain proteins oligomerizing into the amyloid membrane disrupting pore, as exemplified by interactions of the *Podospora anserina* proteins HET-S and HET-s [49]. **D.** Ferroptotic lipid peroxidation in *Magnaporthe oryzae* is attributed to ROS produced by NOXs activated by autophagically released cellular iron (Fe) [51]. MLKL, mixed lineage kinase domain–like; NOX, NADPH oxidase; RCD, regulated cell death; ROS, reactive oxygen species.

Fungi display morphological hallmarks of apoptosis despite sharing only a handful of apoptotic proteins with vertebrates and apparently lacking the entire extrinsic apoptosis pathway [39,40]. Arguably, apoptosis-like cell death (**Fig 1A**) is the most frequently encountered and best understood form of RCD in fungi (for discussion see [41]). In addition to being triggered by antifungal agents with highly diverse modes of action [42] as well as phagocyte generated ROS [43], apoptosis-like RCD can be a consequence of incompatible interactions between nonself isolates in conspecific hyphal fusions [44].

Vegetative/heterokaryon incompatibility during conspecific hyphal fusions is caused by genetic dissimilarities at the *het* loci many of which encode NRL-like proteins [45]. Specifically, coexpression of incompatible alleles at the same locus or incompatible alleles at different loci leads to compartmentalization and cell death of the heterokaryotic portions of the mycelium. The multiplicity of loci regulating self-recognition presents an intriguing possibility that different loci may control distinct modes of cell death. Accordingly, in *Neurospora crassa*, one of the models for studies of vegetative/heterokaryon incompatibility, incompatibilities at loci *het-6* and *het-c* result in apoptotic-like degradation of chromatin and organelles as well as fragmentation of the cytoplasm into small membrane-bound bodies [44,46]. Conversely, incompatibility at the locus *rcd-1* leads to RCD mediated by the plasma membrane-disrupting protein with similarity to animal gasdermin [47] (**Fig 1B**), which in phagocytes contributes to the execution of pyroptosis [48]. In another model, *P*. *anserina*, cell death is induced by activation of the pore-forming protein HET-S by the prion-forming protein HET-s (**Fig 1C**), both encoded at the *het-s* locus [49]. This incompatibility is considered to be a derived form of a more ancient interaction in which conformational changes in the HET-S protein are induced by a nonallelic interaction with the NLR-like protein NWD2, a pattern resembling activation of the pore-forming MLKL, the cell death-execution protein in necroptosis. Also in *P*. *anserina*, nonallelic incompatibility at loci *het-R* and *het-V*, encoding NLR-like proteins, induces vacuolization, autophagy, and cell death [50]. However, as in plants, autophagy appears to play a role in limiting the spread of death signals across the mycelium. Collectively, the involvement of NLR-like proteins in heterokaryon incompatibility–driven RCD of fungi resembles the role of NLRs in mammalian innate immunity responses. In fact, these patterns inspired the hypothesis that in fungi the mechanisms of heterokaryon incompatibility share evolutionary roots with antibacterial defense systems [28]. While this hypothesis remains to be rigorously tested, changes in transcriptional patterns in *P*. *anserina* during heterokaryon incompatible interactions versus co-cultivation with antagonistic bacteria suggest that the expression of genes encoding the NRL-like proteins is favored mainly during heterokaryon incompatibility [28].

Apoptosis-like and heterokaryon incompatibility–driven cell death are not the only forms of RCD deployed by fungi. *Magnaporthe oryzae* relies on developmental cell death of germinating conidia during appressorium formation in rice blast disease [51,52]. This developmental death of fungal cells shares with animal cells hallmarks of autophagy and **ferroptosis** (**Fig 1D**). Ferroptosis is an iron-dependent oxidative cell death involving peroxidation of lipids, resulting in membrane damage [53]. Importantly, in vertebrates, ferroptosis can be triggered by autophagic destruction of iron-storing ferritin, leading to the increase in cellular iron levels [54]. A similar pattern of autophagy modulating the availability of intracellular iron and thus regulating ferroptosis is observed in *M*. *oryzae* [51].

The structural and functional similarities between suicidal programs of fungi and animals suggest shared evolutionary origins of these mechanisms. Given such common roots as well as functional similarities between immune defenses of fungi and animals, we hypothesize that fungi deploy RCD in response to bacterial antagonists.

## 5. A model system to study whether RCD is involved in fungal innate immunity

Recently, the mutualism between *R*. *microsporus* (Mucoromycotina) and *Mycetohabitans* endobacteria (Betaproteobacteria) has emerged from research on fungal-bacterial symbioses as a model for innate immunity studies in fungi [9]. In this symbiosis, endobacteria are inherited from one fungal generation to the next. The 2 partners, the host and endobacteria, can be separated, cultivated independently, and modified genetically [55,56]. Importantly, not all *R*. *microsporus* strains harbor endobacteria. Asymbiotic nonhost strains interact antagonistically with endobacteria isolated from the host and resist being infected, displaying innate immunity defense responses, including oxidative burst [9]. This network of interactions creates a superb framework for elucidating the mechanisms of innate immunity in fungi and testing whether fungi employ RCD for clearing bacteria from their cells. The *Rhizopus* model has several advantages over models representing other fungal lineages, such as *P*. *anserina*. Specifically, Mucoromycotina display a dearth of NLR-like proteins [11–13] and a limited reliance on secondary metabolites for antibacterial warfare [9]. These 2 features are expected to limit antibacterial defenses of Mucoromycotina to the prototypical core of responses that do not rely on NLR-like proteins and secondary metabolites. At the same time, Mucoromycotina are capable of deploying apoptosis-like RCD [57], indicating that they possess the molecular machinery underlying evolutionarily ancient forms of RCD. Importantly, Mucoromycotina are significant and exceedingly difficult to treat opportunistic pathogens of humans and causal agents of mucormycosis [58]. Therefore, understanding the mechanisms of their putative immune RCD is likely to contribute to developing novel antifungal therapies [59]. Such advances would be analogous to the application of statins as adjunctive therapy in mucormycosis [60]. Statins are antihypercholesterolemia drugs inducing apoptosis-like death in Mucoromycotina.

### Outlook

Historically, the oversized significance of secondary metabolites in fungal interactions with bacteria overshadowed the more subtle and intimate aspects of relationships between these organisms. Only now are these aspects becoming apparent thanks to recent discoveries of endosymbiotic bacteria residing in fungal hyphae and spores. Examination of such symbioses revealed that fungi are capable of defending their cellular integrity from invasions by bacteria perceived as antagonists. These antibacterial defenses resemble functionally innate immunity barriers of animals and plants. Fungi also share with animals several genetically encoded cell death functionalities for targeted cell removal. Because of these common patterns, it is likely that fungi employ immune RCD as an ultimate mechanism for eliminating bacterial intruders. We propose to test this hypothesis in the symbiotic system of *R*. *microsporus* and *Mycetohabitans* bacteria. We also anticipate that unraveling the mechanisms of immune RCD in fungi will contribute to developing novel antifungal therapies.

## References

[1] Brubaker SW, Bonham KS, Zanoni I, Kagan JC. Innate immune pattern recognition: a cell biological perspective. Annu Rev Immunol. 2015;33:257–90. doi: 10.1146/annurev-immunol-032414-112240 25581309 PMC5146691

[2] Couto D, Zipfel C. Regulation of pattern recognition receptor signalling in plants. Nat Rev Immunol. 2016;16:537–52. doi: 10.1038/nri.2016.77 27477127

[3] Mermigka G, Amprazi M, Mentzelopoulou A, Amartolou A, Sarris PF. Plant and animal innate immunity complexes: Fighting different enemies with similar weapons. Trends Plant Sci. 2020;25:80–91. doi: 10.1016/j.tplants.2019.09.008 31677931

[4] Jones JD, Vance RE, Dangl JL. Intracellular innate immune surveillance devices in plants and animals. Science. 2016;354:aaf6395. doi: 10.1126/science.aaf6395 27934708

[5] Galluzzi L, Vitale I, Aaronson SA, Abrams JM, Adam D, Agostinis P, et al. Molecular mechanisms of cell death: recommendations of the Nomenclature Committee on Cell Death 2018. Cell Death Differ. 2018;25:486–541. doi: 10.1038/s41418-017-0012-4 29362479 PMC5864239

[6] Washburn JD, Bird KA, Conant GC, Pires JC. Convergent evolution and the origin of complex phenotypes in the age of systems biology. Int J Plant Sci. 2016;177:305–18.

[7] Soanes DM, Talbot NJ. Comparative genome analysis reveals an absence of leucine-rich repeat pattern-recognition receptor proteins in the kingdom Fungi. PLoS ONE. 2010;5:e12725. doi: 10.1371/journal.pone.0012725 20856863 PMC2939053

[8] Keller NP. Translating biosynthetic gene clusters into fungal armor and weaponry. Nat Chem Biol. 2015;11:671–7. doi: 10.1038/nchembio.1897 26284674 PMC4682562

[9] Lastovetsky OA, Krasnovsky LD, Qin X, Gaspar ML, Gryganskyi AP, Huntemann M, et al. Molecular dialogues between early divergent fungi and bacteria in an antagonism versus a mutualism. mBio. 2020;11:e02088–20. doi: 10.1128/mBio.02088-20 32900811 PMC7482071

[10] Xu XL, Lee RTH, Fang HM, Wang YM, Li R, Zou H, et al. Bacterial peptidoglycan triggers *Candida albicans* hyphal growth by directly activating the adenylyl cyclase Cyr1p. Cell Host Microbe. 2008;4:28–39. doi: 10.1016/j.chom.2008.05.014 18621008

[11] Dyrka W, Lamacchia M, Durrens P, Kobe B, Daskalov A, Paoletti M, et al. Diversity and variability of NOD-like receptors in fungi. Genome Biol Evol. 2014;6:3137–58. doi: 10.1093/gbe/evu251 25398782 PMC4986451

[12] Uehling J, Deveau A, Paoletti M. Do fungi have an innate immune response? An NLR-based comparison to plant and animal immune systems. PLoS Pathog. 2017;13:e1006578. doi: 10.1371/journal.ppat.1006578 29073287 PMC5658179

[13] Van der Nest MA, Olson A, Lind M, Vélëz H, Dalman K, Brandström Durling M, et al. Distribution and evolution of *het* gene homologs in the basidiomycota. Fungal Genet Biol. 2014;64:45–57. doi: 10.1016/j.fgb.2013.12.007 24380733

[14] Daskalov A, Habenstein B, Martinez D, Debets AJM, Sabaté R, Loquet A, et al. Signal transduction by a fungal NOD-like receptor based on propagation of a prion amyloid fold. PLoS Biol. 2015;13:e1002059. doi: 10.1371/journal.pbio.1002059 25671553 PMC4344463

[15] Heller J, Clavé C, Gladieux P, Saupe SJ, Glass NL. NLR surveillance of essential SEC-9 SNARE proteins induces programmed cell death upon allorecognition in filamentous fungi. Proc Natl Acad Sci U S A. 2018;115:E2292–301. doi: 10.1073/pnas.1719705115 29463729 PMC5878007

[16] Ipcho S, Sundelin T, Erbs G, Kistler HC, Newman MA, Olsson S.Fungal innate immunity induced by bacterial microbe-associated molecular patterns (MAMPs). G3 (Bethesda). 2016;6(6):1585–95. doi: 10.1534/g3.116.027987 27172188 PMC4889655

[17] Yang Y, Bazhin AV, Werner J, Karakhanova S. Reactive oxygen species in the immune system. Int Rev Immunol. 2013;32:249–70. 23617726 10.3109/08830185.2012.755176

[18] Nanda AK, Andrio E, Marino D, Pauly N, Dunand C. Reactive oxygen species during plant-microorganism early interactions. J Integr Plant Biol. 2010;52:195–204. 20377681 10.1111/j.1744-7909.2010.00933.x

[19] Campos ML, de Souza CM, de Oliveira KBS, Dias SC, Franco OL. The role of antimicrobial peptides in plant immunity. J Exp Bot. 2018;69:4997–5011. 30099553 10.1093/jxb/ery294

[20] Driouich A, Smith C, Ropitaux M, Chambard M, Boulogne I, Bernard S, et al. Root extracellular traps versus neutrophil extracellular traps in host defence, a case of functional convergence? Biol Rev Camb Philos Soc. 2019;94:1685–700. doi: 10.1111/brv.12522 31134732

[21] Balint-Kurti P. The plant hypersensitive response: concepts, control and consequences. Mol. Plant Pathol. 2019;20:1163–78. doi: 10.1111/mpp.12821 31305008 PMC6640183

[22] Kombrink A, Tayyrov A, Essig A, Stöckli M, Micheller S, Hintze J, et al. Induction of antibacterial proteins and peptides in the coprophilous mushroom *Coprinopsis cinerea* in response to bacteria. ISME J. 2019;13:588–602. doi: 10.1038/s41396-018-0293-8 30301946 PMC6461984

[23] Mitchell KF, Zarnowski R, Andes DR. Fungal super glue: The biofilm matrix and its composition, assembly, and functions. PLoS Pathog. 2016;12:e1005828. doi: 10.1371/journal.ppat.1005828 27685086 PMC5042517

[24] Uribe-Querol E, Rosales C. Phagocytosis: Our current understanding of a universal biological process. Front Immunol. 2020;11:1066–6. doi: 10.3389/fimmu.2020.01066 32582172 PMC7280488

[25] Malinovsky FG, Fangel JU, Willats WGT. The role of the cell wall in plant immunity. Front Plant Sci. 2014;5:178. doi: 10.3389/fpls.2014.00178 24834069 PMC4018530

[26] Anding AL, Baehrecke EH. Autophagy in cell life and cell death. Curr Top Dev Biol. 2015;114:67–91. 26431564 10.1016/bs.ctdb.2015.07.012

[27] Qi H, Xia F-N, Xiao S. Autophagy in plants: Physiological roles and post-translational regulation. J Integr Plant Biol. 2021;63:161–79. doi: 10.1104/pp.20.00470 32324339

[28] Lamacchia M, Dyrka W, Breton A, Saupe SJ, Paoletti M. Overlapping *Podospora anserina* transcriptional responses to bacterial and fungal non self indicate a multilayered innate immune response. Front Microbiol. 2016;7:471. doi: 10.3389/fmicb.2016.00471 27148175 PMC4835503

[29] Osherov N, Yarden O. The cell wall of filamentous fungi. In: Borkovich KA, Ebbole DJ, editors. Cellular and Molecular Biology of Filamentous Fungi. Washington, DC: ASM Press. pp. 224–237. (2010). doi: 10.1128/microbiolspec.FUNK-0034-2016

[30] Coll NS, Epple P, Dangl JL. Programmed cell death in the plant immune system. Cell Death Differ. 2011;18:1247–56. doi: 10.1038/cdd.2011.37 21475301 PMC3172094

[31] Man SM, Karki R, Kanneganti TD. Molecular mechanisms and functions of pyroptosis, inflammatory caspases and inflammasomes in infectious diseases. Immunol Rev. 2017;277:61–75. doi: 10.1111/imr.12534 28462526 PMC5416822

[32] Kayagaki N, Kornfeld OS, Lee BL, Stowe IB, O’Rourke K, Li Q, et al. NINJ1 mediates plasma membrane rupture during lytic cell death. Nature. 2021;591:131–6. 33472215 10.1038/s41586-021-03218-7

[33] Weir A, Hughes S, Rashidi M, Hildebrand JM, Vince JE. Necroptotic movers and shakers: cell types, inflammatory drivers and diseases. Curr Opin Immunol. 2021;68:83–97. doi: 10.1016/j.coi.2020.09.008 33160107

[34] Wang J, Wang J, Hu M, Wu S, Qi J, Wang G, et al. Ligand-triggered allosteric ADP release primes a plant NLR complex. Science. 2019;364:eaav5868. doi: 10.1126/science.aav5868 30948526

[35] Salguero-Linares J, Coll NS. Plant proteases in the control of the hypersensitive response. J Exp Bot. 2019;70:2087–95. doi: 10.1093/jxb/erz030 30715462

[36] Liu Y, Schiff M, Czymmek K, Tallóczy Z, Levine B, Dinesh-Kumar SP.Autophagy regulates programmed cell death during the plant innate immune response. Cell. 2005;121:567–77. 15907470 10.1016/j.cell.2005.03.007

[37] Klim J, Gładki A, Kucharczyk R, Zielenkiewicz U, Kaczanowski S. Ancestral state reconstruction of the apoptosis machinery in the common ancestor of eukaryotes. G3 (Bethesda). 2018;8(8):2121–34. 29703784 10.1534/g3.118.200295 PMC5982838

[38] Dorstyn L, Akey CW, Kumar S. New insights into apoptosome structure and function. Cell Death Differ. 2018;25:1194–208. 29765111 10.1038/s41418-017-0025-z PMC6030056

[39] Shlezinger N, Goldfinger N, Sharon A. Apoptotic-like programed cell death in fungi: the benefits in filamentous species. Front Oncol. 2012;2:97. PMCID: PMC3412994 22891165 10.3389/fonc.2012.00097 PMC3412994

[40] Carmona-Gutierrez D, Bauer MA, Zimmermann A, Aguilera A, Austriaco N, Ayscough K, et al. Guidelines and recommendations on yeast cell death nomenclature. Microbial Cell. 2018;5:4–31. doi: 10.15698/mic2018.01.607 29354647 PMC5772036

[41] Hardwick JM Do fungi undergo apoptosis-like programmed cell death? mBio. 2018;9:e00948–18. 30065087 10.1128/mBio.00948-18 PMC6069115

[42] Sharon A, Finkelstein A, Shlezinger N, Hatam I. Fungal apoptosis: function, genes and gene function. FEMS Microbiol Rev. 2009;33:833–54. 19416362 10.1111/j.1574-6976.2009.00180.x

[43] Shlezinger N, Irmer H, Dhingra S, Beattie SR, Cramer RA, Braus GH, et al. Sterilizing immunity in the lung relies on targeting fungal apoptosis-like programmed cell death. Science. 2017;357:1037–41. 28883073 10.1126/science.aan0365 PMC5628051

[44] Jacobson DJ, Beurkens K, Klomparens KL. Microscopic and ultrastructural examination of vegetative incompatibility in partial diploids heterozygous at het loci in *Neurospora crassa*. Fungal Genet Biol. 1998;23:45–56. 9514694 10.1006/fgbi.1997.1020

[45] Hutchison EA, Glass NL. Programmed cell death and heterokaryon incompatibility in filamentous fungi. In: Witzany G, editor. Biocommunication of Fungi. Dordrecht: Springer. pp. 115–138. (2012). doi: 10.1007/978-94-007-4264-2

[46] Marek SM, Wu J, Glass NL, Gilchrist DG, Bostock RM Nuclear DNA degradation during heterokaryon incompatibility in *Neurospora crassa*. Fungal Genet Biol. 2003;40:126–37. 14516765 10.1016/s1087-1845(03)00086-0

[47] Daskalov A, Mitchell PS, Sandstrom A, Vance RE, Glass NL. Molecular characterization of a fungal gasdermin-like protein. Proc Natl Acad Sci U S A. 2020;117:18600–7. 32703806 10.1073/pnas.2004876117 PMC7414189

[48] Liu X, Zhang Z, Ruan J, Pan Y, Magupalli VG, Wu H, et al. Inflammasome-activated gasdermin D causes pyroptosis by forming membrane pores. Nature. 2016;535:153–8. 27383986 10.1038/nature18629 PMC5539988

[49] Saupe SJ. Amyloid signaling in filamentous fungi and bacteria. Annu Rev Microbiol. 2020;74:673–91. 32689912 10.1146/annurev-micro-011320-013555

[50] Pinan-Lucarré B, Paoletti M, Clavé C. Cell death by incompatibility in the fungus *Podospora*. Semin Cancer Biol. 2007;17:101–11. 17204431 10.1016/j.semcancer.2006.11.009

[51] Shen Q, Liang M, Yang F, Deng YZ, Naqvi NI. Ferroptosis contributes to developmental cell death in rice blast. New Phytol. 2020;227:1831–46. 32367535 10.1111/nph.16636

[52] Eseola AB, Ryder LS, Osés-Ruiz M, Findlay K, Yan X, Cruz-Mireles N, et al. Investigating the cell and developmental biology of plant infection by the rice blast fungus *Magnaporthe oryzae*. Fungal Genet Biol. 2021;154:103562. 33882359 10.1016/j.fgb.2021.103562

[53] Chen X, Comish PB, Tang D, Kang R. Characteristics and biomarkers of ferroptosis. Front Cell Dev Biol. 2021;9:637162. 33553189 10.3389/fcell.2021.637162 PMC7859349

[54] Hou W, Xie Y, Song X, Sun X, Lotze MT, Zeh HJ, et al. Autophagy promotes ferroptosis by degradation of ferritin. Autophagy. 2016;12:1425–8. 27245739 10.1080/15548627.2016.1187366 PMC4968231

[55] Carter ME, Carpenter SCD, Dubrow ZE, Sabol MR, Rinaldi FC, Lastovestsky OA, et al. A TAL effector-like protein of an endofungal bacterium increases the stress tolerance and alters the transcriptome of the host. Proc Natl Acad Sci U S A. 2020;117:17122–9. 32632014 10.1073/pnas.2003857117 PMC7382252

[56] Lax C, Navarro-Mendoza MI, Pérez-Arques C, Navarro E, Nicolás FE, Garre V. Stable and reproducible homologous recombination enables CRISPR-based engineering in the fungus *Rhizopus microsporus*. Cell Reports Methods. 2021;1:100124. 35475217 10.1016/j.crmeth.2021.100124 PMC9017206

[57] Roze LV. Linz JE Lovastatin triggers an apoptosis-like cell death process in the fungus *Mucor racemosus*. Fungal Genet Biol. 1998;25:119–33. 9974223 10.1006/fgbi.1998.1093

[58] Baldin C, Ibrahim AS. Molecular mechanisms of mucormycosis—The bitter and the sweet. PLoS Pathog. 2017;13:e1006408. 28771587 10.1371/journal.ppat.1006408 PMC5542377

[59] Kulkarni M, Stolp ZD, Hardwick JM. Targeting intrinsic cell death pathways to control fungal pathogens. Biochem Pharmacol. 2019;162:71–8. 30660496 10.1016/j.bcp.2019.01.012 PMC6430978

[60] Bellanger AP, Tatara AM, Shirazi F, Gebremariam T, Albert ND, Lewis RE, et al. Statin concentrations below the minimum inhibitory concentration attenuate the virulence of *Rhizopus oryzae*. J Infect Dis. 2016;214:114–21. 26984141 10.1093/infdis/jiw090 PMC5007635

